# Perspectives of the use of Perampanel in Psychiatric Symptoms

**DOI:** 10.1192/j.eurpsy.2025.2070

**Published:** 2025-08-26

**Authors:** J. J. De Frutos Guijarro, B. Macías Gamo, N. E. Chinchurreta De Lora, C. Merino Del Villar, B. Romero Salicio

**Affiliations:** 1 Psychiatry, Hospital Can Misses, Ibiza, Spain

## Abstract

**Introduction:**

Perampanel is a selective antagonist of the AMPA receptor for glutamate, primarily approved for the treatment of certain types of epilepsy. With the evolving understanding of psychiatric disorders’ neurobiology, it’s hypothesized that targeting the glutamatergic system could offer substantial therapeutic benefits (Perversi F, Costa C, Labate A, Lattanzi S, Liguori C, Maschio M, et al. The broad-spectrum activity of perampanel: state of the art and future perspective of AMPA antagonism beyond epilepsy. Front Neurol [Internet]. 2023 [cited 2024 Sep 2] ;14:1182304).

**Objectives:**

The purpose of this study is to evaluate the effectiveness of perampanel in managing psychiatric symptoms such as sleep disturbances, depression, anxiety, and irritability.

**Methods:**

A comprehensive review of scientific studies was carried out, centering on clinical trials and observational research that explored the application of perampanel in individuals exhibiting psychiatric symptoms. The review included articles published between 2013 and 2024, utilizing databases like PubMed, Scopus, and PsycINFO for sourcing. The inclusion criteria covered studies that assessed the impact of perampanel on psychiatric conditions, detailing both the clinical results and any side effects.

**Results:**

Findings indicate that perampanel may have beneficial effects in reducing symptoms of insomnia (Abenza-Abildúa MJ, Suárez-Gisbert E, Thuissard-Vasallo IJ, Andreu-Vazquez C. Perampanel in chronic insomnia. Clin Neurol Neurosurg. 2020 May 1;192), depression and anxiety (Scorrano G, Lattanzi S, Salpietro V, Giannini C, Chiarelli F, Matricardi S. The Cognitive and Behavioural Effects of Perampanel in Children with Neurodevelopmental Disorders: A Systematic Review. J Clin Med [Internet]. 2024 Jan 10 [cited 2024 Sep 3];13(2)) in certain patient groups. However, significant adverse effects were also reported, including behavioural changes and increased aggression in some cases, necessitating careful monitoring during treatment.

**Conclusions:**

Numerous antiepileptic medications have been effectively utilized in treating psychiatric conditions. Perampanel, in particular, has demonstrated effectiveness in managing nocturnal seizures, preserving sleep architecture, and treating restless legs syndrome. A study conducted in Spain revealed that combining perampanel with either an antidepressant or an anxiolytic significantly enhances sleep quality after three months in patients without epilepsy (Abenza-Abildúa MJ, Suárez-Gisbert E, Thuissard-Vasallo IJ, Andreu-Vazquez C. Perampanel in chronic insomnia. Clin Neurol Neurosurg. 2020 May 1;192).

While there are no large-scale clinical trials specifically targeting mood disorders, some ongoing research is exploring the broader psychiatric effects of Perampanel, including its impact on anxiety disorders.

**Disclosure of Interest:**

None Declared

